# Supplementary material for: Determinants of knowledge, attitude, and practice among patients with type 2 diabetes mellitus: A cross-sectional multicenter study in Tanzania
Source: PLOS Glob Public Health. 2023 Dec 1;3(12):e0001351. doi: 10.1371/journal.pgph.0001351 (PMC10691713; doi:10.1371/journal.pgph.0001351)
Supplement: Attachment — Submitted filename: Responses to review comments.docx [file pgph.0001351.s001.docx]

**Responses to review comments**

| **Comment** | **Review** | **Page/section** |
| --- | --- | --- |
| **Editors’ comment** |  |  |
| The language is poor, and the paper needs detailed editing for syntax, please. | We have worked on the issue of language including improvement of grammar, structure as well as syntax | NA |
| There are major methodological flaws in this paper from the sample size calculation to the questionnaires used. | All methodological flaws including issues questionnaire used for data correction have been appropriately worked on. For stance, we have elaborated clearly on how we obtained the sample size  Regarding the questionnaire we used, we explained that we validated it by calculating a Chronban Alpha after we have conducted the pilot study. Also, we modified some difficult words including translating them into Swahili words for easy understanding | Page 6, under data collection method and tools |
| **Title:**  Why was general diabetes KAP not assessed; why is it limited to complications as stated in the title and Aims? This does not come through in the Introduction | After revising, we modified the title so as to include the general KAP for diabetes and not limit KAP to diabetes complications as it was in the first submitted manuscript | Title of the study |
| **Introduction** What is the current prevalence of diabetes in Tanzania?  o not provide arbitrary examples that are not relevant to this study population e.g., Bangladesh vs. Slovenia | We included the current data regarding diabetes in Tanzania | Page 3, under introduction section |
| **Methods** Using diabetes prevalence (data not presented) for the sample size calculation does not accord with the study aim to determine KAPs. This is a major methodological flaw. | This was made clear in the corrected version by elaborating that the total sample size was obtained conveniently, that is every diabetic patient who met inclusion criteria was sampled until the sample size was met | Page 5, under-sampling method |
| ‘…study population of 1,255’ – this seems incorrect. | This was a typing error and it was removed in the corrected version |  |
| What standardized international questionnaires were used to assess knowledge, attitudes and practices? Why was a questionnaire from Ethiopia use? Has it been validated in Tanzania? Was the questionnaire translated into the local languages? The authors need to provide more details on the questionnaire/questions. | We decided to use the questionnaire from the previous study done in Ethiopia because that study has adapted more than one validated questionnaire, to make it valid in the Tanzania setting, we validated it by calculating Cronbach Alpha so as to obtain internal consistency for enhancing reproducibility. We did so by conducting a pilot study after we have translated into the Swahili language | Page 4, under data collection method and tools |
| What specific factors related to knowledge, attitudes and practices were examined? It is vital to include such detail. | We close examiner the three domains (knowledge, attitude, and practice) by running an association by a number of independent factors including socidemographic factors, co-morbidities, and knowledge in the case of attitude and practice. This helps us to establish the specific factors related to the three domains | Page 13, 15, and 17 under the results section (tables 3,4, and 5) |
| What co-morbidities were evaluated? | The co-morbidities evaluated were HIV, hypertension, and PUD | Page 6, under the data collection section |
| Discussion The main findings of the study, in line with the study Aim, should be summarized in the first paragraph. Unfortunately, the current 1st paragraph is inappropriate. | We summarised the main finding of the study according to the objective of the study in the first paragraph of the discussion | Page 18, under discussion of the study |
| **Reviewer #1:** |  |  |
| The study is good one but linguistic framework is poor. After corrected those it may be resubmitted. | We appreciate the comments given by the reviewer also, we thank the reviewer for his constructive comments which warrant the resubmission of the work after making major changes. We have critically revised the linguistic framework including correction of grammar, structure, and typos | NA |
| The major flaws are in the abstract and introduction. | All the major flaws in the abstract and introduction were corrected as indicated in the resubmitted manuscript. This includes an example of rephrasing the context of the abstract as well as making changes for some of the sentences in its methodological part | Page 2, abstract section |
| Discussion needs to be revised. | We revised the discussion as it was commented on by the reviewer including the addition of the first paragraph on the key findings of the study as reflected by the aims | Page 18, under discussion section |
| **Reviewer #2: Reviewer Comment:** |  |  |
| The prevalence of Diabetes is increasing worldwide, especially in developing countries like Asia and Africa. In this context, this multi centric study from Tanzania is very crucial. | We are so much grateful for the comments given by the reviewer | NA |
| The study design and the power of the study are satisfactory. However, there is a selection bias in the inclusion criteria- patients who denied to sign the consent were excluded. This is understandable but whether the patients who denied permission belong to a particular group (socio-economic status) has to be looked into in detail. It is also important to rule out if the patients selected have previously received diabetes education. This is because the KAP status of patients who have received diabetes education will be high. This aspect has not been specified in the sampling criteria | This is very observation. However, in our case, those who denied signing the informed consent did not below to any specific group. For example socio, cultural or religious groups. Also, in order to make the study population homogenous and not heterogeneous all those who had prior knowledge of diabetes were excluded | Page 5, under-sampling method |
| The observation that despite good knowledge and attitude, the practice domain remains considerably low is often noticed. The suggestion that the factors affecting practice improvement has to be identified and improved is appreciated. | We are so gracious for this observation made by the reviewer. | NA |
| Minor errors like spelling and grammatical mistakes should also be looked into. | To the best of our understanding, we have comprehensively revised the manuscript and therefore all typos and grammatical issues have been corrected | NA |
| As an attempt to determine the KAP status of Type 2 Diabetes, this study is significant and can be recommended for publication. | We thank the reviewer for the good comment. | NA |
