# Supplementary material for: Determinants of knowledge, attitude, and practice among patients with type 2 diabetes mellitus: A cross-sectional multicenter study in Tanzania
Source: PLOS Glob Public Health. 2023 Dec 1;3(12):e0001351. doi: 10.1371/journal.pgph.0001351 (PMC10691713; doi:10.1371/journal.pgph.0001351)
Supplement: Attachment — Submitted filename: POINT TO POINT RESPONSES TO REVIEW COMMENTS.docx [file pgph.0001351.s003.docx]

Reviewer #1: 
1. Under the methodology section, study participants exclusion criteria the statement "Additionally, we excluded all patients who had prior knowledge on diabetes so as to make the study population homogeneous". How was this implemented? What types of knowledge measurements the authors used to assess? A written informed consent should be used even before the pilot study (prior knowledge assessment) despite 20 pilot study were conducted. In addition, the knowledge discrepancy is a useful determinant for policy makers with having the possible association. Hence, the authors should elaborate the statement as exclusion criteria is a key for study participants selection.

**Response from authors: The comment raised by the reviewer is very important. In this study we aimed to investigate a homogeneous study population so that we could avoid bias. In order to do so, all subjects who agreed to have been involved or participated in sessions for raising of awareness and knowledge on diabetes such as education sessions when attending diabetes clinic as well as community campaign regarding awareness of diabetes. Most of studies done did not consider the issue of homogeneity for the level of knowledge and awareness regarding diabetes.**

2. The study was conducted at eight study sites (three district hospitals and five regional referral hospital). I have a doubt for the selection methods of the study sites as the authors responded "because of here are no studies addressing knowledge, attitude and practice in patients with T2DM from the study areas in Tanzania." This implies there are data mentioning other health institution concerning the KAP assessment. Additionally, the population is not correctly sampled with an appropriate sampling techniques (formula). This is the heart of the study. If sampling frame is not appropriately stated, the study will not represent the population.

**Response from authors: Thank you so much for a very good observation. Regarding selection of the study sites, it is true that we selected the study sites conveniently; meaning that we did not apply any of the probability sampling method. This is because we needed a large sample size as much as we could. This was based on the possible population size we were able to observe after making a survey before conducting the study. The use of non-probability method has been used also in other previous studies regarding the subject on KAP. For example, Fatema et al. Knowledge attitude and practice regarding diabetes mellitus among Nondiabetic and diabetic study participants in Bangladesh. BMC Public Health (2017) 17:364 DOI 10.1186/s12889-017-4285-9,**

3. Under "Data collection method and tools" section, with referencing the Ethiopian research as a tool for measurements of KAP. However, the referenced Ethiopian article is not mentioning knowledge. The article was only assessed the attitude and practice with three section (Socio-demographic status, attitude, and practice) You can access the study from (<https://bmcpublichealth.biomedcentral.com/articles/10.1186/s12889-020-08953-6#additional-information>). Hence, the authors should use another knowledge measurement tools or update their reference.

**Response from authors: Thank you for a nice comment from the reviewer: We added another study from Malaysia regarding questions on assessment of level of knowledge among study participants (page 5, last paragraph)**

Reviewer #2: Thank you to the authors for submitting their manuscript.
1. Abstract needs to be revised as it can be more precise and irrelevant details can be removed

**Response from authors: The abstract was revised to make it precise and irrelevant details were removed.**

2. In the abstract it is mentioned that level of knowledge is significantly associated with attitude and practice. However, no statistical results are shown and discussed in the manuscript

**Response from authors: statistical results were shown by indicating the p-value for the associated values. Also, in the revised manuscript we included points in the discussion regarding association of level of knowledge with altitude (page 18, paragraph 2).**

3. “The reason for selecting these study sites is that there are no studies addressing knowledge, attitude and practice in patients with T2DM from the study areas in Tanzania” this line is repeated in the STUDY AREA section. Please revise this.

**Response from authors: The repeated line at the section of study area was deleted in the revised manuscript.**

4. The study excluded the patients with prior knowledge, hence, selecting the sample of individuals with low level of knowledge. It will cause the magnitudes to be biased downwards resulting in biased estimates. Due to the randomization the chances of bias are minimized but excluding patients with knowledge will disrupt the randomization process. Also, convince sampling is not the most suitable to obtain population representative sample

**Response from authors: Thank you very much for a nice comment. “By prior knowledge on diabetes”, we referred to a group of diabetics who reported to have participated in education sessions when attending diabetes clinic as well as community campaign regarding awareness of diabetes. By including this group of patients with other patients who reported to have not had participated in programmes or education sessions which help in raising awareness among diabetics and the population in general, this would make the study population heterogeneous and not homogeneous. This would also cause bias in assessing level of knowledge among study participants due to lack of homogeneity of the participants.**

5. In MATERIALS AND METHODS section, there is no mention of how the sample size of 979 was calculated and through which methodology. Please provide the appropriate details in this section.

**Response from authors: Thank you for a nice comment. It is indicated in the manuscript that we used convenience sampling method, meaning that all subjects who met the inclusion criteria during the study period were recruited simultaneously until the sample size needed was met. We wanted a possible large sample size, therefore, by employing any of the probability sampling and would lead to small sample size because other subjects despite meeting the inclusion criteria would not be included in the analysis.**

6. In the discussion section needs revision. There are weak linkages between knowledge, attitude and practice in the manuscript

**Response from authors: Thank you for a very good comment. We revised the whole discussion and we corrected the section by improving on the issue of reasoning and explanation so as to make it clear to the readers.**

7. A lot of repetition in the discussion section. Please revise it.

**Response from authors: All repetitions were removed in the discussion section to make the discussion precise.**

8. In the conclusion, it was written that, attitude is not influenced by knowledge, however, in the abstract it was written that knowledge significantly associated with both attitude and practice. Also, no statistical test or values are shown in the paper regarding the association between knowledge, attitude and practice.

**Response from authors: It was written that level of knowledge was associated with altitude in both abstract and conclusion section of the main text. Also, we indicated the p-values for the associated values in the result sections of both abstract and main text (page 2 and page 11-14).**

9. Apart from Chi-square test and why was no regression analysis performed for knowledge, attitude and practice with the socio-demographic variable collected?

**Response from authors: We understand the concern raised by the reviewer. Our objective was to assess for the factors associated with Knowledge, Attitude, and Practices towards diabetes. This is the reason we used Chi-square test to assess the factors associated with Knowledge, Attitude, and Practices. We did not use logistic regression analysis because our objective was not to determine predictors of KAP.**

10. Overall, the writing of the paper needs significant improvements and the word count of the paper should be reduced.

**Response from authors: the whole manuscript was critically revised and typographical and grammatical errors were corrected.**

11. Lastly, there are a lot of repetition in the paper which needs to be adjusted.

**Response from authors: All repetitions were corrected in the whole manuscript after making critical review.**
