# Supplementary material for: Determinants of knowledge, attitude, and practice among patients with type 2 diabetes mellitus: A cross-sectional multicenter study in Tanzania
Source: PLOS Glob Public Health. 2023 Dec 1;3(12):e0001351. doi: 10.1371/journal.pgph.0001351 (PMC10691713; doi:10.1371/journal.pgph.0001351)
Supplement: Attachment — Submitted filename: POINT TO POINT FOR REVIEW COMMENTS (1).docx [file pgph.0001351.s004.docx]

**POINT TO POINT RESPONSES TO REVIEWERS’ COMMENTS**

1. Please review your reference list to ensure that it is complete and correct. If you have cited papers that have been retracted, please include the rationale for doing so in the manuscript text, or remove these references and replace them with relevant current references. Any changes to the reference list should be mentioned in the rebuttal letter that accompanies your revised manuscript. If you need to cite a retracted article, indicate the article’s retracted status in the References list and also include a citation and full reference for the retraction notice.

**Response: all the references were cross-checked thoroughly and they are all well-aligned (page 19-23).**

**Reviewer #3**

Dear authors, I am reviewing the current version of the manuscript and have noticed that you have already made substantial revisions in response to comments from previous reviewers. I have the following comments:

1) The language of the manuscript needs to be improved considerably. I will suggest that you ask someone with good command of English to do a proofreading and copyediting to improve the clarity of the language in the paper.

**Response: thank you very much for a very good comment. The whole manuscript has been revised to improve the English language. All typographic errors, grammatical errors, repetitions, and punctuation errors have been corrected.**

2) In the Introduction section, please revise "The International Federation of Diabetes (IDM)" to "The International Diabetes Federation (IDF)"

**Response: we have changed the words “The International Federation of Diabetes (IDM)" to "The International Diabetes Federation (IDF)" (page 1, paragraph 3).**

3) In the Introduction section, there is a sentence - "This tremendous and alarming drastic increase in the incidence of DM in developing countries in which Tanzania is included has been linked to substantial demographic change from traditional ways of living to Westernized and urbanized [3]". Please share the link for reference 3, as I would like to check whether the reference is appropriate or not.

**Response: Thank you for a good observation. We have corrected the reference which is relevant for the information provided (Chiwanga FS, Njelekela MA, Diamond MB, Bajunirwe F, Guwatudde D, Nankya-Mutyoba J, et al. Urban and rural prevalence of diabetes and pre-diabetes and risk factors associated with diabetes in Tanzania and Uganda. Glob Health Action 2016;9(1):1–8) (page 19, reference 3) and not the other one which we have deleted.**

4) Out of the 8 study sites, how many were public hospitals and how many were private?

**Response: we have indicated in the revised manuscript that only one health facility (Haydom district hospital) is the faith-based health facility, and the rest were public (page 4, paragraph 3)**

5) How was the sample size of 979 calculated?

**Response: Response: thank you for a nice comment. we have clearly indicated in the revised manuscript calculation of the sample size (page 5, paragraph 2)**

6) There are two sentences in the Results section which I could not understand - "Study participants who were working in private health facilities had significantly higher percentage of adequate knowledge (79.5%) than those working in the health facilities owned by government (58.7%) (p<0.001)." and "The percentage of study participants with positive attitude working at referral health facility was significantly higher (53.2%) than that of study participants who were working at either district (50.3%) or regional health facility (44.8%) (p = 0.008) (Table 4)." Do you mean that the participants of the study were working at the health facilities? Are they not supposed to be patients who were visiting these health facilities for their treatment?

**Response:** **were apologize for the confusion. We have corrected the statement to make it clear (page 2, abstract section)**

7) **In the Discussion section, please explain why you decided to exclude all patients who reported to have been involved or participated in sessions for raising of awareness and knowledge on diabetes from the study, and what are the implications of this. Are there any other studies which have used a similar exclusion criteria? Is this to understand the knowledge, attitude and practices of the naive population? Is it to understand the kind of efforts that the Ministry of Health has to undertake in order to sensitize this vulnerable segment of the population and to assess what are the key messages to be incorporated in social and behavior change communication (SBCC) campaigns for this population?**

**Response:** Most studies have included even participants who have been involved in knowledge and awareness-raising campaigns or training. For the current study, we thought there is a need of excluding patients who had previous exposure in the training for raising awareness and knowledge so we understand the knowledge, attitude, and practices of the naive population (vulnerable population) in order to understand the kind of efforts that the Ministry of Health has to undertake in order to sensitize this vulnerable population

8) In the 3rd paragraph of the Discussion section, there is a sentence - "Furthermore, it has been found that patients who stay longer with T2DM have a significant high level of knowledge compared to patients with short duration of the disease". Please revise the language to "patients who were earlier diagnosed with diabetes" and "patients who were recently diagnosed with diabetes" or similar terminology

**Response: the statement was changed according to the suggestion or advice from the reviewer (page 15, paragraph 3).**

9) The first sentence in the Conclusion section is grammatically incorrect.

**Response: we appreciate very much for a nice comment. we have worked on this comment by correcting the first statement in the conclusion section and also improve the whole section of conclusion (page 18, paragraph 2).**

10) Reference 2 should be "International Diabetes Federation". Please correct the spelling of diabetes.

**Response: the word “diabetes” was written correctly in the reference number 2 (page 19).**

11) Please fix the style of Reference 15.

**Response: reference number 15 was referenced or written correctly (page 21).**

12) In the Abstract, there is a sentence which I found confusing - "Working at a district hospital was significantly associated with adequate knowledge (p<0.01), positive attitude (p<0.008), and appropriate practice (p<0.004)." This is similar to comment #6. Are the participants in your study the employees of the health facilities or the patients seeking care at these facilities? Since you have used "working at a district hospital", it implies that staff of a district hospital are more likely to have adequate knowledge, positive attitude and appropriate practice. But that does not make sense.

**Response: were apologize for the confusion. We have corrected the statement to make it clear (page 2, abstract section).**

**Reviewer #4**

Thank you for the opportunity to review the manuscript by Joho, et al., who must be commended for their work on this hugely important topic.

1. Is the manuscript presented in an intelligible fashion and written in standard English? That science has to be communicated in the English language unfortunately disadvantages non-native speakers. Nonetheless, the manuscript’s standard of English could be much improved. Manuscript currently has grammatical errors, repetitions, and does not read easily.

**Response: we have worked hard to improve the English language by correcting all grammatical errors, repetitions, and typographical errors. We therefore think the flow of ideas and readability of the paper has been improved.**

2. Has the statistical analysis been performed appropriately and rigorously? Authors state that they undertook both descriptive and inferential analyses. However, their results are largely, if not solely, descriptive. Analyses reported in Tables 3-5 do not warrant the interpretations or conclusions that are drawn/made in the manuscript. For example, authors claim that “Positive attitude was increasing linearly with increasing level of education of study participants…” [p.41, Association of sociodemographic characteristics with attitude]. Chi-square tests (which were undertaken by authors) assess for overall differences in expected vs observed frequencies; they are not trend tests, which is what authors imply in much of their discussion of results. The previous reviewer`s suggestion that authors use regression modeling for their inferential analyses was not needed. Results from inferential analyses are best reported with their corresponding 95% CIs.

**Response: Thank you for a very good observation.** **Based on your comment number 5, we have changed the analysis by performing binary logistic regression analysis for determining the predictors of knowledge, attitude, and practice (Table 3, 4 and 5 from page 10-13). All Tables that were showing Chi-square test have been deleted.**

3. Under “Sampling Method”, authors state that “…participants were consecutively recruited until the required total sample size of 979 was met.” However, sample size estimation is not reported.

**Response: thank you for a nice comment. we have clearly indicated in the revised manuscript calculation of the sample size (page 5, paragraph 2)**

4. Tables 2-5 in fact report the prevalence (or frequency) of “adequate knowledge”, “good attitude” and “appropriate practices”, respectively. Thus, these tables could be much simplified, and easier to understand, if only the “yes” outcome (of a dichotomous variable [yes/no]) were reported versus reporting both “yes” and “no” outcomes.

**Response: Tables 3, 4, and 5 have been changed to Tables indicating logistic regression analysis based on the comment given in number 5 below (Table 3, 4 and 5 from page 10-13). However, we kindly request for Table 2 to remain as it is because it provides more comprehensive information.**

5. Reporting results of (unadjusted and adjusted) regression modeling in Tables 3-5 will be more informative than the descriptive summaries that are presently presented in these tables.

**Response: we have performed binary logistic regression analysis so as to provide more informative findings as it was recommended (Table 3, 4 and 5 from page 10-13).**

6. In summary, the authors` manuscript might be much improved if the assistance of a statistician were sought.

**Response: for the sake of improving the English language of the paper, we have revised the whole manuscript and all typos, punctuation errors and grammatical errors were corrected.**
