# Supplementary material for: Determinants of knowledge, attitude, and practice among patients with type 2 diabetes mellitus: A cross-sectional multicenter study in Tanzania
Source: PLOS Glob Public Health. 2023 Dec 1;3(12):e0001351. doi: 10.1371/journal.pgph.0001351 (PMC10691713; doi:10.1371/journal.pgph.0001351)
Supplement: Attachment — Submitted filename: PGPH-D-22-01621R3 - Knowledge, attitude and practice towards type 2 diabetes mellitus.docx [file pgph.0001351.s005.docx]

July 10, 2023

The Editor,

**RE: Manuscript PGPH-D-22-01621R3 - Determinants of Knowledge, Attitude, and Practice among Patients with Type 2Diabetes Mellitus: a Multicenter Study in Tanzania**

The manuscript by Joho, *et al.,* refers. Overall, their results and conclusions represent an important contribution to the subject particularly as they come from an understudied and under-reported part of the world.

However, some revisions will be required and the below suggestions may be worth considering.

1. **Language**

While considerable progress has been made in improving the grammar, the language of the manuscript can still be considerably improved and made more concise.

One example is the simultaneous use of past and present tense *, “This study* ***aims to assess*** *the levels of knowledge…. This* ***was a*** *cross-sectional multicenter hospital-based study which* ***included****…”* [Abstract] . The assistance of someone with good command should be sought.

2. **Statistics/Data analysis**

Further improvements will be helpful in the reporting/presentation of the results. Examples include:

Sample size estimation – The Leslie Kish formula, as I understand it, is meant for calculating effective sample sizes in weighted surveys. Authors should ensure they have correctly applied this.

Table 1 – Including mean (SD) or median (IQR) for age and duration of illness. This is stated in the “Data analysis” section but not reported. Also, frequency and percentage need not be two separate columns but one column “frequency (%)”.

Table 2 – These results can be better presented as a bar graph/plot with 95% CI spikes.

Tables 3, 4, 5 – It is customary to place the reference category (1.00) for any variable at the top, and not the bottom. Consistency in use of decimal numbers in reporting results will be helpful.

3. **Discussion**

The “Discussion” section can be made considerably shorter and more concise. In its current form, it largely reads as another literature review or “Background” section. A discussion of the implications of these results in the Tanzanian context will be more relevant and informative.

4. **References**

Reference #2, in the Introduction section, suggests that Tanzania`s T2DM prevalence increased from 5.7% to 12.3% in a space of **2 years**, from 2019 to 2021. This seems unlikely, an authors should double check this.
