# Supplementary material for: Determinants of knowledge, attitude, and practice among patients with type 2 diabetes mellitus: A cross-sectional multicenter study in Tanzania
Source: PLOS Glob Public Health. 2023 Dec 1;3(12):e0001351. doi: 10.1371/journal.pgph.0001351 (PMC10691713; doi:10.1371/journal.pgph.0001351)
Supplement: Attachment — Submitted filename: Response to reviewer.docx [file pgph.0001351.s006.docx]

**Response to reviewer’s comments**

| **Comments** | **Response** | **Page no, section** |
| --- | --- | --- |
| 1. **Language**   While considerable progress has been made in improving the grammar, the language of the manuscript can still be considerably improved and made more concise.  One example is the simultaneous use of past and present tense *, “This study* ***aims to assess*** *the levels of knowledge…. This* ***was a*** *cross-sectional multicenter hospital-based study which* ***included****…”* [Abstract] . The assistance of someone with good command should be sought. | Thank you for a good observation, we have corrected the use of past and present tense simultaneously throughout the manuscript including the abstract. The whole manuscript has been critically revised and we believe the readability has been significantly improved | Whole document |
| 2. **Statistics/Data analysis**   Further improvements will be helpful in the reporting/presentation of the results. Examples include:  Sample size estimation – The Leslie Kish formula, as I understand it, is meant for calculating effective sample sizes in weighted surveys. Authors should  ensure they have correctly applied this. | The reason why we used the Kish-Leslie formula in calculating sample size is that the Kish-Leslie formula is suitable for calculating the proportion of a single population. This study also included a single population of patients with type diabetes mellitus | Not applicable |
| Table 1 – Including mean (SD) or median (IQR) for age and duration of illness. This is stated in the “Data analysis” section but not reported. Also, frequency and percentage need not be two separate columns but one column “frequency (%)”. | We appreciate so much the observation made by the reviewer. We have added the mean and standard deviation that we miss for the illness's duration as a continuous variable.  We have modified Table 1 by combining frequency and percentage in one column as it was suggested by the reviewer | Page 8,9, paragraph one |
| Table 2 – These results can be better presented as a bar graph/plot with 95% CI spikes. | We have taken the reviews’ comment, and we have changed the table to figure | Page 10 |
| Tables 3, 4, 5 – It is customary to place the reference category (1.00) for any variable at the top, and not the bottom. Consistency in use of decimal numbers in reporting results will be helpful. | We understand the concern raised by the reviewer. However, based on the experience from published articles, it is true that the positioning of the reference group depends on the intention of the researchers in comparing the groups of the given variable to the outcome of interest. This means the reference group can either be at the or bottom among groups of a variable | Not applicable |
| 3. **Discussion**   The “Discussion” section can be made considerably shorter and more concise. In its current form, it largely reads as another literature review or “Background” section. A discussion of the implications of these results in the Tanzanian context will be more relevant and informative. | We are so much grateful concerning these comments, we have rephrased a number of sentences in different paragraphs so as to shorten and make the precise discussion as it was requested by the reviewer | Discussion section |
| 4. **References**   Reference #2, in the Introduction section, suggests that Tanzania`s T2DM prevalence increased from 5.7% to 12.3% in a space of **2 years**, from 2019 to 2021. This seems unlikely, an authors should double check this. | We acknowledge the reviewer’s comment, we have reviewed the reference | References |
